# Dr. Stokes on Fever

**Published:** 1854-03

**Authors:** 


### DR. STOKES ON FEVER.

---

Dr. Stokes admits that his opinions in reference to fever have undergone a great change since he first became a lecturer on the Practice of Medicine. After describing the former practice, in a lecture recently delivered in the Meath Hospital, Dublin, he says:

"The treatment of fever is reducible to a simple formula. We cannot cure fever; no man has ever cured fever. Fever in this sense may be called incurable, because we don't know how to cure it. It is, however, curable by itself. If you leave it to its own course, it will often cure itself. The great object, then, is to preserve the patient's life from the dangers which threaten him, pending the existence of this condition of fever—of this special state of life. If you can keep him alive to the fourteenth day, or the eighteenth day, or the twenty-first day, he will recover. What are the dangers that occur in these cases? One I have already pointed out to you—it is the danger of debility; the other is the danger arising from the accidental secondary affections. We cure the patient by preventing him from dying. We lead him on to that period when by the extraordinary law of the disease, it will itself spontaneously terminate. We prevent him from dying of exhaustion, by food, by the use of stimulants, and by tonics. We save him from the danger which the local diseases may expose him to, by meeting them as early as we can discover them. And herein lies the whole secret of the treatment of fever—to preserve the patient, at the least expense to his constitution, up to the time when, by the natural law, the disease will spontaneously subside.

